# Radiological Cardiothoracic Ratio as a Potential Predictor of Right Ventricular Enlargement in Patients with Suspected Pulmonary Embolism Due to COVID-19

**DOI:** 10.3390/jcm10235703

**Published:** 2021-12-04

**Authors:** Krystian Truszkiewicz, Małgorzata Poręba, Rafał Poręba, Paweł Gać

**Affiliations:** 1 Center for Diagnostic Imaging, University Clinical Hospital in Wrocław, Borowska 213, PL 50-556 Wroclaw, Poland; ktruszkiewicz@usk.wroc.pl; 2 Department of Paralympic Sports, Wroclaw University of Health and Sport Sciences, Witelona 25a, PL 51-617 Wroclaw, Poland; poreba1@wp.pl; 3 Department of Internal Medicine, Occupational Diseases and Hypertension, Wroclaw Medical University, Borowska 213, PL 50-556 Wroclaw, Poland; rafal.poreba@umw.edu.pl; 4 Centre for Diagnostic Imaging, 4th Military Hospital, Weigla 5, PL 50-981 Wroclaw, Poland; 5 Department of Population Health, Division of Environmental Health and Occupational Medicine, Wroclaw Medical University, Mikulicza-Radeckiego 7, PL 50-368 Wroclaw, Poland

**Keywords:** cardiothoracic ratio, chest radiograph, computed tomography angiography, COVID-19, pulmonary embolism, right ventricular enlargement

## Abstract

The aim of the study was to determine the usefulness of the radiological cardiothoracic ratio (CTR) as a predictor of right ventricular enlargement in patients with suspected pulmonary embolism during COVID-19. The study group consisted of 61 patients with confirmed COVID-19, suspected of pulmonary embolism based on physical examination and laboratory tests (age: 67.18 ± 12.47 years). Computed tomography angiography (CTA) of pulmonary arteries and chest radiograph in AP projection with cardiothoracic ratio assessment were performed in all patients. Right ventricular enlargement was diagnosed by the ratio of right ventricular to left ventricular (RV/LV) dimensions in pulmonary CTA with two cut-off points: ≥0.9 and ≥1.0. Heart silhouette enlargement was found when CTR on the chest radiograph in the projection AP > 0.55. The mean values of RV/LV and CTR in the studied group were 0.96 ± 0.23 and 0.57 ± 0.05, respectively. Pulmonary embolism was diagnosed in 45.9%. Right ventricular enlargement was documented in 44.3% or 29.5% depending on the adopted criterion RV/LV ≥ 0.9 or RV/LV ≥ 1.0. Heart silhouette enlargement was found in 60.6%. Patients with confirmed pulmonary embolism (PE+) had a significantly higher RV/LV ratio and CTR than patients with excluded pulmonary embolism (PE−) (RV/LV: PE+ 1.08 ± 0.24, PE− 0.82 ± 0.12; CTR: PE+ 0.60 ± 0.05, PE− 0.54 ± 0.04; *p* < 0.05). The correlation analysis showed a statistically significant positive correlation between the RV/LV ratio and CTR (r = 0.59, *p* < 0.05). Based on the ROC curves, CTR values were determined as the optimal cut-off points for the prediction of right ventricular enlargement (RV/LV ≥ 0.9 or RV/LV ≥ 1.0), being 0.54 and 0.55, respectively. The sensitivity, specificity, and accuracy of the CTR criterion >0.54 as a predictor of RV/LV ratio ≥0.9 were 0.412, 0.963, and 0.656, respectively, while those of the CTR criterion >0.55 as a predictor of RV/LV ratio ≥1.0 were 0.488, 0.833, and 0.590, respectively. In summary, in patients with suspected pulmonary embolism during COVID-19, the radiographic cardiothoracic ratio can be considered as a prognostic factor for right ventricular enlargement, especially as a negative predictor of right ventricular enlargement in the case of lower CTR values.

## 1. Introduction

The virus SARS-CoV-2 causes a complex of symptoms of a viral respiratory infection, in severe cases causing acute respiratory failure and death [1]. During the pandemic, patients started demonstrating numerous complications other than strictly respiratory ones, e.g., concerning the nervous, vascular, and cardiopulmonary system [2,3]. The literature provides numerous reports regarding a series of cardiovascular complications secondary to COVID-19 [4,5]. Well-recognized cardiac manifestations of COVID-19 are heart damage secondary to cardiac ischemia and/or myocardial infarction as well as myocarditis [6,7,8]. Others include arrhythmias, cardiogenic shock and cardiomyopathy [6]. Another issue related to the COVID-19 infection is the frequent occurrence of pulmonary embolism and deep vein thrombosis, more common than in the course of other viral infections (e.g., the H1N1 flu virus) [9,10]. Meta-analysis demonstrated respectively 16.5% and 14.8% occurrence of acute pulmonary embolism and deep vein thrombosis in patients infected with COVID-19, whereas more than a half of the patients with pulmonary embolism did not suffer from deep vein thrombosis [11].

In its natural course, pulmonary embolism causes more or less intensive overload of the right cardiac ventricle. It is estimated that about 45% patients with acute pulmonary embolism will develop right ventricular insufficiency [12], and as much as 3.8% of them will develop chronic thromboembolic pulmonary hypertension [13]. The right ventricular insufficiency develops because of increased secondary load caused by pulmonary artery occlusion. Once the compensation mechanisms of the right ventricle are exceeded, the right ventricle becomes enlarged and the right ventricular projection is further decreased, leading to a reduced supply of the myocardium with oxygenated blood and ischemia [14].

Apart from the pulmonary embolism, the right ventricular enlargement may cause pulmonic valve stenosis, pulmonary hypertension, arterial and/or ventricular septal defects, tricuspid valve regurgitation, hypertrophic cardiomyopathy, congenital defects, etc. An assessment of the RV size and functions can be made via echocardiography, computed tomography and magnetic resonance [15,16,17]. Although very useful, in COVID-19 infected patients, the echocardiography methods should not be used routinely, due to the risk of transmitting the infection to the personnel. On the other hand, high concurrence of CT and MR measurements is emphasized [16,17].

A test of choice, characterized by high sensitivity and specificity for diagnosing acute pulmonary embolism is pulmonary artery computed tomography angiography (CTA) [18]. Apart from assessment of the pulmonary embolism itself, the test can be used to evaluate RV enlargement, the morphology of the interventricular septum, etc. An additional, important parameter that can be specified in the pulmonary artery CTA is the RV/LV ratio. This parameter is defined as the ratio of the maximum RV dimension to the corresponding LV dimension, measured from endocardium to endocardium on axial CTA scans, which are the closest to a four-chamber projection, or a reconstructed four-chamber projection. Values of more than 0.9 are considered abnormal [19], and they signify a considerably higher risk of an adverse course of the embolism. It is emphasized that this ratio is highly sensitive (about 92%) for the evaluation of right ventricle insufficiency, and it was considered an independent predictor for a poor prognosis in PE patients [20]. The value of this ratio in the risk stratification for PE patients is also emphasized by the 2019 guidelines issued by ESC and ERS, concerning the diagnostics and treatment in acute pulmonary embolism [21]. Additionally, a large meta-analysis, also cited by the aforesaid guidelines, indicates that a ratio of RV/LV ≥ 1.0 involves a 2.5-fold increase in the risk of death for any reason and a 5-fold increase in the risk of PE-related death [22].

The cardiothoracic ratio is an easy to calculate indicator in the assessment of myocardial enlargement. It is defined as the ratio of the largest transverse heart dimension to the largest transverse chest dimension, measured to the internal rib surface on a chest radiograph [23]. Any value above 0.50 is considered incorrect and may be a sign of cardiomyopathy [24]. Values > 0.55 were considered incorrect for radiographs in the AP projection [25,26], which, in relation to the health condition of patients hospitalized with acute respiratory failure secondary to COVID-19, is the basic radiographic projection.

The purpose of the study was to determine the usefulness of the cardiothoracic ratio (CTR) as a predictor of right ventricular enlargement in patients with suspected pulmonary embolism secondary to COVID-19.

## 2. Materials and Methods

The study group consisted of 61 patients with confirmed COVID-19, in which pulmonary embolism was suspected based on a physical examination and laboratory tests. Group size was determined using a sample size calculator. The selection conditions were as follows: population size 2 million, fraction size 0.2, maximum error 10%, confidence level 95%. The required minimum size of the study group was 61. The criteria of inclusion in the study were as follows: age ≥ 18 years, a positive result of a nasopharynx smear test against SARS-CoV-2 (detected presence of the N2 gene for SARS-CoV-2 and/or the E gene for *Betacoronaviridae* using the qRT-PCR method), an increased concentration of D-dimers in the blood, a clinically indicated CTA of the pulmonary arteries and an AP chest radiograph performed within 48 h. The criteria of exclusion in the study were as follows: an ambiguous result of the pulmonary artery CTA scan, a diagnosed pulmonary or mediastinal carcinoma, large amounts of fluid in the pleural cavity/cavities, fluid present in the pericardial sac. The anthropometric parameters of the study group of patients are presented in Table 1.

The studies were performed in one clinic treating patients hospitalized for COVID-19 and its complications. The study was performed in the first half of 2021.

At subsequent stages, the study group was divided into subgroups, following the criteria of age, BMI, gender, enlarged heart silhouette in chest radiograph as well as enlarged right ventricle, and diagnosed pulmonary embolism in the CTA. The criteria for distinguishing the study subgroups and the sizes of the subgroups are listed in Table 2.

The study was performed as part of the research project titled “Radiologic cardiothoracic ratio as a predictor for the size of the heart estimated via echocardiography, computer tomography and magnetic resonance”, approved by the local bioethical committee (KB-414/2021).

The methodology of the study comprised an analysis of the basic anthropometric parameters and the following diagnostic images: AP chest radiograph (CR) and computed tomography angiography (CTA) of the pulmonary arteries.

According to the adopted criteria of inclusion in the study, the interval between the pulmonary artery CTA and the AP chest radiograph did not exceed 48 h. In case more than one CR was acquired within 48 h before or after the pulmonary artery CTA, the analysis was performed using the chest radiograph acquired within the shortest interval from the pulmonary artery CTA.

On account of the clinical condition of the patients, chest radiographs were acquired in the lying position, in the anterior–posterior projection (AP), using a bedside X-ray machine. Radiographs were acquired, as far as possible, while the patient was holding their breath, with maximum inhalation, using the X-ray lamp set at 120 kV.

**Figure 1:**
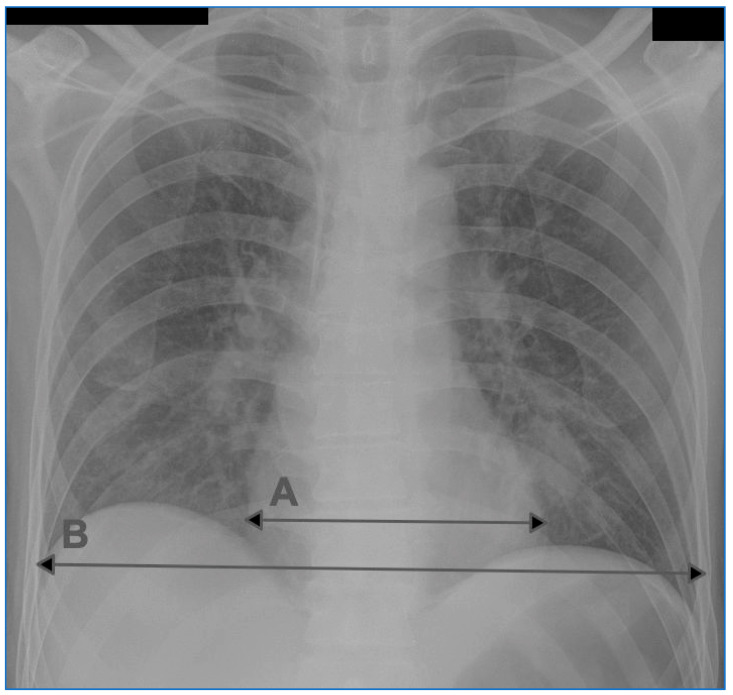
An example of CTR measurement on a chest radiograph in the AP projection. A: transverse dimension of the heart silhouette, B: transverse dimension of the chest.

The cardiothoracic ratio (CTR) was assessed using a diagnostic station conforming to the legal regulations for radiologic tests. The maximum cardiac width (C width) and the maximum thoracic width (T width) were measured. The value of the cardiothoracic ratio was calculated using the following formula: CTR = C width/T width. Enlargement of the heart silhouette was diagnosed if, in the AP chest radiograph, the CTR > 0.55. An example of the CTR measurement in the AP chest radiograph is presented in Figure 1.

CTAs of the pulmonary arteries were acquired using a 64-slice computed tomography scanner SOMATOM Definition AS+ (Siemens Healthcare, Erlangen, Germany), in accordance with the standard protocol. In this protocol, the sequence of actions was as follows: tomograph, pre-monitoring and monitoring with the ROI set within the pulmonary trunk/left pulmonary artery at the tracheal bifurcation level, and the proper acquisition with a start triggered by the contrast saturation of 100 HU within the ROI. The acquisition encompassed the area from the pulmonary apices to the costophrenic angles. The exposure kilovolt value was 120 units, with variable mAs values. The scans were performed using intravenous contrast with a constant volume of 60 mL non-ionic contrast, infused with an automatic syringe into the cubital fossa veins, at the infusion rate of 4.0 mL/s. Basic reconstructions were made in axial planes, in 3.0 mm and 0.75 mm slices, along with secondary reconstructions MPR and MIP in frontal and sagittal planes.

**Figure 2:**
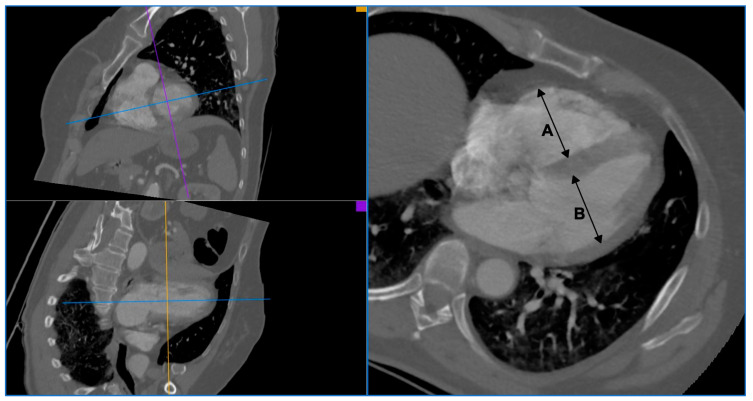
An example of RV/LV ratio measurement in CTA of pulmonary arteries. A: right ventricle diameter, B: left ventricle diameter.

The pulmonary artery CTA images required for this analysis were assessed using an application for post-processing of computed tomography images, syngo.CT Cardiac Function (Siemens Healthcare, Erlangen, Germany), by two staff radiologists experienced in the assessment of cardiac and vascular angiography images. Pulmonary embolism (PE+) was diagnosed in cases where filling defects in the pulmonary arteries were found. To assess right ventricle enlargement, the size of both ventricles was measured. The size of the right and left ventricle (RV diameter and LV diameter) was measured based on a multiplanar reconstruction (MPR), in a four-chamber projection, perpendicularly to the long axis of the ventricles, at 1/3 distance between the mitral valve and the apex. The diameter of a ventricle was recognized as the distance between the endocardium of the free wall of the ventricle and the endocardium of the interventricular septum. The papillary muscles were included in the lumen of the chamber. Each time, the average measurement result was considered the final RV diameter and LV diameter values. At the time of the measurement, the radiologists assessing the ventricle dimension had no knowledge of the CTR value. Enlargement of the right ventricle was diagnosed based on the ratio of the right and left ventricle size (RV/LV) in the pulmonary artery CTA, using two different cut-off points: ≥0.9 and ≥1.0. An example of measuring the RV/LV ratio in the pulmonary artery CTA is presented in Figure 2.

Statistical analysis was performed using the Dell Statistica 13 (Dell Inc., Tulsa, OK, USA) application. For quantitative variables, arithmetic means and standard deviations were calculated. The Shapiro–Willke test was used to verify normal distribution of the variables. Quantitative independent variables with normal distribution were further analyzed using a t test for independent variables. Variables with distribution other than normal were analyzed using the U Mann–Whitney test for independent quantitative variables. Results for the qualitative variables were expressed as a percentage. Qualitative variables were analyzed using the chi-square test. Correlation was analyzed to specify the relationship between the analyzed variables. Pearson correlation coefficients were determined for quantitative variables with normal distribution, and Spearman correlation coefficients for quantitative variables with distribution other than normal. Moreover, the accuracy was tested, with proposed cut-off points for the tests estimated based on the ROC (receiver operating characteristic) curves. The adopted statistical significance level was *p* < 0.05.

## 3. Results

The average CTR value in the study group of the patients was 0.57 ± 0.05. Radiological enlargement of the heart silhouette was diagnosed in 60.6% of the subjects. Based on the CTA of the pulmonary arteries, pulmonary embolism was diagnosed in 45.9% of the subjects. The average RV/LV ratio was 0.96 ± 0.23. Right ventricle enlargement, depending on the adopted criterion of RV/LV ≥ 0.9 or RV/LV ≥ 1.0, was documented in 44.3% or 29.5% of subjects. The results of the heart silhouette measurement by a chest radiograph and the results of the analyzed variables in the pulmonary artery CTA in the examined group of patients with suspected pulmonary embolism secondary to COVID-19 are presented in Table 3.

A comparative analysis of the subgroups divided on the basis of the CTR cut-off point of 0.55 demonstrated that the patients with enlarged heart silhouette were characterized by statistically significantly higher values of the RV/LV in the pulmonary artery CTA than the patients with non-enlarged heart silhouette; they also significantly more often met the criteria of right ventricle enlargement (both defined as RV/LV ≥ 0.9 and as RV/LV ≥ 1.0). The selected variables of the pulmonary artery CTA in the study subgroups divided on the basis of the criterion of enlarged heart silhouette in the chest radiograph are presented in Table 4.

When comparing the subgroups divided on the basis of the cut-off points RV/LV ≥ 0.9 and RV/LV ≥ 1.0, it was documented that the patients with an enlarged right ventricle had statistically significantly higher CTR values than those with non-enlarged right ventricles. In patients with enlarged right ventricle, the heart silhouette enlargement (defined as CTR > 0.55 in the AP chest radiograph) was observed statistically significantly more often than in patients with non-enlarged right ventricle. The size of the heart silhouette in CR of study subgroups divided on the basis of the criterion of right ventricle enlargement in the pulmonary artery CTA is presented in Table 4.

In a comparison of the subgroups divided on the basis of diagnosed pulmonary embolism, it was found that the patients with confirmed pulmonary embolism had statistically significantly higher CTR and RV/LV than those with excluded pulmonary embolism. In the patients with confirmed pulmonary embolism, enlarged heart silhouette was found statistically significantly more often than in those with excluded pulmonary embolism. These study subgroups demonstrated no differences in the frequency of enlarged right ventricle in the pulmonary artery CTA. The size of the heart silhouette in CR and the selected variables of the pulmonary artery CTA in the study subgroups divided on the basis of the criterion of pulmonary embolism diagnosed in the pulmonary artery CTA are presented in Table 4.

**Figure 3:**
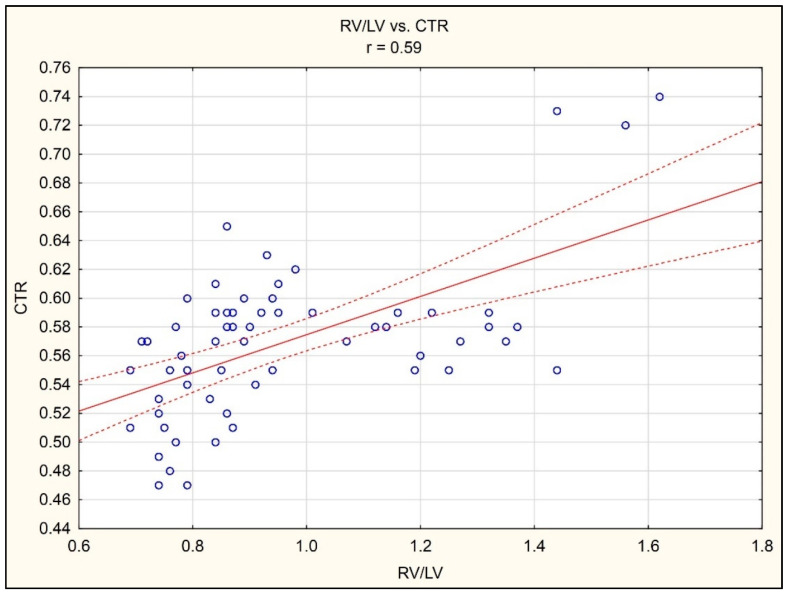
Correlation between RV/LV ratio in CTA of pulmonary arteries and CTR in chest radiograph in the AP projection.

A correlation analysis demonstrated the existence of a statistically significant positive correlation between RV/LV and CTR (r = 0.59, *p* < 0.05), (Figure 3).

The demonstrated correlation occurred in all the divided subgroups, except for the group with excluded pulmonary embolism. The results of an analysis of the heart silhouette size in the chest radiograph and the size of the right ventricle in the pulmonary artery CTA in the whole study group and study subgroups are presented in Table 5.

**Figure 4:**
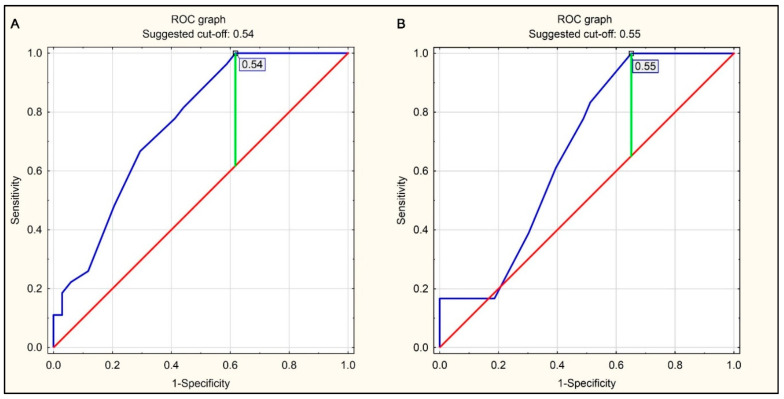
ROC curves for predicting right ventricular enlargement (RV/LV ratio ≥ 0.9 or RV/LV ratio ≥ 1.0 in pulmonary CTA) using the CTR value on the chest radiograph in the AP projection. (**A**) Right ventricular enlargement defined as RV/LV ≥ 0.9 in pulmonary CTA. (**B**) Right ventricular enlargement defined as RV/LV ≥ 1.0 in pulmonary CTA.

The ROC curves were used to determine CTR values constituting optimal cut-off points for the prediction of right ventricle enlargement (RV/LV ≥ 0.9 or RV/LV ≥ 1.0), amounting to 0.54 and 0.55, respectively (Figure 4A,B).

The sensitivity, specificity, and accuracy of CTR > 0.54 as a predictor for RV/LV ≥ 0.9 were 0.412, 0.963, and 0.656, respectively, while those of CTR > 0.55 as a predictor for RV/LV ≥ 1.0 were 0.488, 0.833, and 0.590, respectively. The test accuracy analysis indicated that the predictors had a significantly higher specificity than sensitivity. The results of the accuracy analysis for the radiological cardiothoracic ratio as a predictor of right ventricle enlargement in the pulmonary artery CTA for the whole study group are presented in Table 6.

Table 6 present also the results of an analysis performed in the divided subgroups. The radiological cardiothoracic ratio with the highest accuracy is a predictor of right ventricle enlargement in the pulmonary artery CTA in the subgroups of females, overweight/obese patients, and patients aged ≥ 71 years.

## 4. Discussion

COVID-19 has become an enormous epidemiological problem for the entire world; we witnessed as numerous health care systems in many countries around the globe collapsed one by one. Apart from acute respiratory failure secondary to pneumonia, this illness involves numerous complications, including cardiovascular ones. In 28 out of the 61 patients in the investigated group, the pulmonary artery CTA scan demonstrated the presence of pulmonary embolism (45.9%), which confirms the weight of the problem in patients hospitalized with diagnosed COVID-19. Similar percentages of pulmonary embolism were reported by researchers from Italy (44.7%) [27] and London (46.2%) [28] for their respective groups. Right ventricular failure is one of the main problems in this patient group.

Dimensioning of the right ventricle is reflected in the guidelines regarding the diagnostics and treatment of pulmonary embolism, and the RV/LV ratio may be a prognostic factor [21]. To assess advanced pneumonia, patients admitted to hospitals often undergo a chest radiograph, usually AP, on account of their health condition. When interpreting these radiographs, it is possible to determine the cardiothoracic ratio, which does not prolong the interpretation task in any significant way. Earlier studies already demonstrated that the CTR could be a prognostic factor for myocardial enlargement in various illnesses [29,30,31,32]. Values >0.55 were considered incorrect for radiographs in the AP projection [25,26], and the same value was demonstrated in a study investigating the dependence between CTR in the AP projection and heart measurements in computed tomography [33].

According to our data analysis, such determined CTR can be considered as a prognostic factor for right ventricular enlargement in patients with pulmonary embolism secondary to COVID-19. RV/LV was significantly higher in patients with enlarged heart silhouette than in patients with non-enlarged heart silhouette. CTR was significantly higher in patients with an enlarged right ventricle (higher RV/LV) than in patients with non-enlarged right ventricles (lower RV/LV). There was a positive correlation between RV/LV and CTR. In addition, the accuracy of CTR > 0.54 as a predictor for RV/LV ≥ 0.9 was 65.6%. However, considering that the specificity of prediction is much higher than its sensitivity (over 80% compared to less than 50%), especially lower CTR values may indicate a lack of right ventricular enlargement in patients with COVID-19. The high sensitivity of prediction always indicates the usefulness of the test in confirming a given state, while the high specificity of prediction indicates the usefulness of the test in excluding a given state.

The research published so far also confirms our observations, although the number of these studies is small. Researchers from Iran proved that CTR determined during a chest CT is a strong predictor for mortality in patients with COVID-19, and its values grow together with the affected volume of the pulmonary parenchyma. Just like us, the researchers demonstrated that over half of the patients had increased CTR; in our group it was 60.6% [34]. Considering the lack of any statistically significant difference between CTR determined on a chest radiograph and in computed tomography [35], one can conclude that a clinically indicated radiograph could be one of the screening factors in the evaluation of right ventricle enlargement; also, a study on the association between the CTR and the ventricle size in patients with systolic and diastolic heart failure demonstrated a higher relationship between the CTR and RV than LV size [36].

Researchers from Warsaw reached different conclusions, when they failed to prove a significant CTR value in predicting the right ventricle size in a different group of patients, namely those after a Fallot tetralogy correction [37]. The same applies to the general population of minors with heart defects [38]; it was demonstrated that in healthy children the correlation between the CTR and the size of the heart chambers determined via echocardiography was small [39]. This merits the question of how the surgery and the defect itself affected the shape and consequently the size of the heart silhouette and chest in the radiograph, and did it affect the CTR measurements? Another research work that negated a significant added value of the CTR in the assessment of the right ventricle size was a study on the relation between these parameters in patients with restrictive pulmonary diseases (pure restrictive ventilatory impairment) [40]. In this study, the researchers observed only a small correlation between the CTR and enlarged right ventricle (this study was undoubtedly limited by the size of the study group, which was only 19 patients). The studies performed on the adult population referred to in this paragraph emphasized the lack of any added value provided by the estimation of RV size on a lateral radiograph.

The Iranian researchers, while indicating significantly lower value to estimating heart size using CTR than using echocardiography, at the same time emphasized the prevalence of the radiographic method and cited it as a good, low-cost screening assessment for heart silhouette enlargement [41].

Our study was encumbered by several limitations, which still fail to significantly reduce the value of the results. In terms of the studied group, the limitations included the small size of the study group, the lack of data on pharmacotherapy for chronic diseases, as well as the inability to perform analyses in subgroups of patients with specific comorbidities (due to the insufficient size of the subgroups, which would be created based on the criteria for the occurrence of subsequent comorbidities). The results of the present study are a starting point for further studies on a larger group of patients, which will allow consideration for the importance of comorbidities, as well as their pharmacotherapy. In terms of the research methodology itself, the following limitations should be mentioned. The study did not consider the volume of the pulmonary parenchyma affected by the inflammation, while from a pathophysiological perspective, an increase in the affected volume of the pulmonary parenchyma would increase the afterload of the right ventricle, which may cause an enlargement of the right ventricle, and, in consequence, the CTR. Therefore, these data should be related to the volume of the lungs affected by the inflammation, particularly in the group of patients without diagnosed pulmonary embolism, but with a diagnosed enlargement of the right ventricle or CTR. Additionally, determination of the CTR on an AP radiograph has numerous limitations. Due to the health condition of the patients in the study group, it was not possible to perform a PA radiograph; therefore, it appears that comparing the CTR measurement to the CTR determined in pulmonary CTA could provide added value. Moreover, we had no access to chest radiographs or chest CT scans of the patients that were taken before current hospitalization. It seems that excluding patients with prior CTR and RA/LV increases from the study group could improve the sensitivity and specificity of the assessment for the investigated parameters in the context of pulmonary embolism secondary to COVID-19. Finally, the pulmonary artery CTA was only performed on patients with suspected pulmonary embolism instead of the whole group, making it impossible to determine the number of patients with clinically silent pulmonary embolism.

## 5. Conclusions

In patients with suspected pulmonary embolism secondary to COVID-19, the radiological cardiothoracic ratio can be considered as a prognostic factor for right ventricular enlargement. Considering that the specificity of prediction is much higher than its sensitivity, especially lower CTR values may indicate a lack of right ventricular enlargement in patients with COVID-19.

## Figures and Tables

**Table 1:** General characteristics of the study group.

	X	SD
age [years]	67.18	12.47
BMI [kg/m^2^]	28.11	3.84
	**%**	**n**
gender		
men	63.9	39
women	36.1	22
age		
<60 years	16.4	10
≥60 years	83.6	51
body mass		
normal	22.9	14
overweight	50.8	31
obesity	26.2	16
comorbidities		
a history of myocardial infarction	14.7	9
a history of stroke	13.1	8
arterial hypertension	24.6	15
peripheral arterial disease	9.8	6
diabetes	11.5	7
a history of cancer	13.1	8
COPD	14.7	9
asthma	4.9	3
stomach and duodenal ulcers	4.9	3
chronic kidney disease	3.3	2
hypothyroidism/hyperthyroidism	8.2	5
osteoporosis	13.1	8

BMI—body mass index; COPD—chronic obstructive pulmonary disease; n—number; SD—standard deviation; X—mean.

**Table 2:** Criteria for distinguishing the study subgroups.

Differentiating Variable	Selection Criterion	Subgroup	Size of the Subgroup
age	median age (71 years)	A: ≥71 years B: <71 years	A: 33 B: 28
BMI	upper limit of the normative value (25 kg/m^2^)	C: overweight/obesity (≥25 kg/m^2^) D: normal body mass (<25 kg/m^2^)	C: 47 D: 14
gender		E: men F: women	E: 39 F: 22
enlargement of the heart silhouette	cardiothoracic ratio (CTR) on chest radiograph in antero-posterior projection > 0.55	G: enlarged heart silhouette (CTR > 0.55) H: non-enlarged heart silhouette (CTR ≤ 0.55)	G: 37 H: 24
enlargement of the right ventricle	right ventricle diameter to left ventricle diameter ratio (RV/LV) in CTA ≥ 0.9	I: enlarged right ventricle (RV/LV ≥ 0.9) J: non-enlarged right ventricle (RV/LV < 0.9)	I: 27 J: 34
	right ventricle diameter to left ventricle diameter ratio (RV/LV) in CTA ≥ 1.0	K: enlarged right ventricle (RV/LV ≥ 1.0) L: non-enlarged right ventricle (RV/LV < 1.0)	K: 18 L: 43
pulmonary embolism	presence of embolic material in pulmonary arteries on CTA	M: confirmed pulmonary embolism N: excluded pulmonary embolism	M: 28 N: 33

BMI—body mass index; CTA—computed tomography angiography.

**Table 3:** The size of the heart silhouette in the chest radiograph and selected variables in the CTA examination of the pulmonary arteries in the study group.

	X	SD
C width [mm]	189.10	27.61
T width [mm]	331.59	33.61
CTR	0.57	0.05
RV diameter [mm]	49.55	14.33
LV diameter [mm]	51.66	8.77
RV/LV	0.96	0.23
	**%**	**n**
CTR > 0.55	60.6	37
RV/LV ≥ 0.9	44.3	27
RV/LV ≥ 1.0	29.5	18
PE+	45.9	28

C width—transverse dimension of the heart silhouette; CTR—cardiothoracic ratio; LV—left ventricle; PE+—pulmonary embolism; RV—right ventricle; T width—transverse dimension of the chest.

**Table 4:** The size of the heart silhouette in the chest radiograph and selected variables in the CTA examination of the pulmonary arteries in the study subgroups. (A) Selected variables of pulmonary artery CTA in the study subgroups divided according to the criterion of cardiac enlargement in the chest radiograph. (B) The size of the heart silhouette in the chest radiograph in the study subgroups divided according to the criterion of right ventricular enlargement in the CTA of pulmonary arteries (if RV/LV ≥ 0.9 defines right ventricular enlargement). (C) The size of the heart silhouette in the chest radiograph in the study subgroups divided according to the criterion of right ventricular enlargement in the CTA of pulmonary arteries (if RV/LV ≥ 1.0 defines right ventricular enlargement). (D) The size of the heart silhouette in the chest radiograph and selected variables of the CTA of the pulmonary arteries in the study subgroups divided according to the criterion of pulmonary embolism in the CTA of the pulmonary arteries.

A	Enlarged Heart Silhouette: CTR > 0.55 (Subgroup G, n = 37)		Non-Enlarged Heart Silhouette: CTR ≤ 0.55 (Subgroup H, n = 24)		*p*
	X	SD	X	SD	
RV diameter [mm]	53.12	15.42	44.04	10.53	0.014
LV diameter [mm]	51.65	9.43	51.67	7.83	0.994
RV/LV	1.03	0.24	0.85	0.18	0.004
	**%**	**n**	**%**	**n**	
RV/LV ≥ 0.9	59.5	22	20.8	5	0.003
RV/LV ≥ 1.0	40.5	15	12.5	3	0.019
PE+	54.0	20	33.3	8	0.057
**B**	**Enlarged Right Ventricle:** **RV/LV ≥ 0.9** **(Subgroup I, n = 27)**		**Non-Enlarged Right Ventricle:** **RV/LV < 0.9** **(Subgroup J, n = 34)**		***p***
	**X**	**SD**	**X**	**SD**	
C width [mm]	198.80	24.65	181.39	27.73	0.013
T width [mm]	333.56	31.60	330.03	35.51	0.688
CTR	0.60	0.05	0.55	0.04	<0.001
	**%**	**n**	**%**	**n**	
CTR > 0.55	81.5	22	44.1	15	0.003
**C**	**Enlarged Right Ventricle:** **RV/LV ≥ 1.0** **(Subgroup K, n = 18)**		**Non-Enlarged Right Ventricle:** **RV/LV < 1.0** **(Subgroup L, n = 43)**		***p***
	**X**	**SD**	**X**	**SD**	
C width [mm]	199.11	28.16	184.91	26.59	0.067
T width [mm]	332.00	31.56	331.42	34.79	0.951
CTR	0.60	0.06	0.56	0.04	0.003
	**%**	**n**	**%**	**n**	
CTR > 0.55	83.3	15	51.2	22	0.019 *
**D**	**Confirmed Pulmonary Embolism** **(Subgroup M, n = 28)**		**Excluded Pulmonary Embolism:** **(Subgroup N, n = 33)**		***p***
	**X**	**SD**	**X**	**SD**	
C width [mm]	199.49	26.06	176.85	24.53	0.001
T width [mm]	334.09	33.64	328.64	33.94	0.533
CTR	0.60	0.05	0.54	0.04	<0.001
RV diameter [mm]	56.04	15.32	41.89	8.09	<0.001
LV diameter [mm]	51.88	9.43	51.39	8.09	0.831
RV/LV	1.08	0.24	0.82	0.12	<0.001
	**%**	**n**	**%**	**n**	
CTR > 0.55	85.7	24	39.4	13	0.002
RV/LV ≥ 0.9	57.1	16	33.3	11	0.062
RV/LV ≥ 1.0	39.3	11	21.2	7	0.122

* Statistical significance; C width—transverse dimension of the heart silhouette; CTR—cardiothoracic ratio; LV—left ventricle; PE+—pulmonary embolism; RV—right ventricle; T width—transverse dimension of the chest.

**Table 5:** Correlation of the size of the heart silhouette in the chest radiograph and the size of the right ventricle in the CTA of the pulmonary arteries in the study group and subgroups.

Group/Subgroup	RV/LV vs. CTR Correlation	
	Correlation Coefficient (r)	*p*
whole study group	0.59	<0.001
subgroup A: ≥71 years	0.61	<0.001
subgroup B: <71 years	0.55	0.002
subgroup C: overweight/obesity (BMI ≥ 25 kg/m^2^)	0.61	<0.001
subgroup D: normal body mass (BMI < 25 kg/m^2^)	0.58	0.029
subgroup E: men	0.44	0.005
subgroup F: women	0.78	<0.001
subgroup G: enlarged heart silhouette (CTR > 0.55)	0.52	0.001
subgroup H: non-enlarged heart silhouette (CTR ≤ 0.55)	0.42	0.039
subgroup I: enlarged right ventricle (RV/LV ≥ 0.9)	0.47	0.013
subgroup J: non-enlarged right ventricle (RV/LV < 0.9)	0.44	0.010
subgroup K: enlarged right ventricle (RV/LV ≥ 1.0)	0.67	0.002
subgroup L: non-enlarged right ventricle (RV/LV < 1.0)	0.56	<0.001
subgroup M: confirmed pulmonary embolism	0.43	0.012
subgroup N: excluded pulmonary embolism	0.29	0.127

CTR—cardiothoracic ratio; LV—left ventricle; RV—right ventricle.

**Table 6:** Sensitivity, specificity, and accuracy of the radiographic cardiothoracic ratio as a predictor of right ventricular enlargement in pulmonary CTA. (A) In the whole group. (B) In subgroups divided according to the age criterion. (C) In subgroups divided according to the BMI criterion. (D) in subgroups divided according to the gender criterion. (E) in subgroups divided according to the pulmonary embolism criterion.

A	Prediction of Right Ventricular Enlargement (RV/LV ≥ 0.9)		Prediction of Right Ventricular Enlargement (RV/LV ≥ 1.0)	
CTR value that is the optimal cut-off point for prediction based on the ROC curve	>0.54		>0.55	
sensitivity	0.412		0.488	
specificity	0.963		0.833	
accuracy	0.656		0.590	
positive predictive value	0.933		0.875	
negative predictive value	0.565		0.405	
likelihood ratio of a positive result	11.118		2.930	
likelihood ratio of a negative result	0.611		0.614	
**B**	**Subgroup A: ≥71 Years**		**Subgroup B: <71 Years**	
	**Prediction of Right Ventricular Enlargement** **(RV/LV ≥ 0.9)**	**Prediction of Right Ventricular Enlargement** **(RV/LV ≥ 1.0)**	**Prediction of Right Ventricular Enlargement** **(RV/LV ≥ 0.9)**	**Prediction of Right Ventricular Enlargement** **(RV/LV ≥ 1.0)**
CTR value that is the optimal cut-off point for prediction based on the ROC curve	>0.55	>0.55	>0.58	>0.58
sensitivity	0.556	0.455	0.875	0.810
specificity	0.933	0.909	0.417	0.429
accuracy	0.727	0.606	0.679	0.714
**C**	**Subgroup C: Overweight/Obesity** **(BMI ≥ 25 kg/m** **^2^** **)**		**Subgroup D: Normal Body Mass** **(BMI < 25 kg/m^2^)**	
	**Prediction of Right Ventricular Enlargement** **(RV/LV ≥ 0.9)**	**Prediction of Right Ventricular Enlargement** **(RV/LV ≥ 1.0)**	**Prediction of Right Ventricular Enlargement** **(RV/LV ≥ 0.9)**	**Prediction of Right Ventricular Enlargement** **(RV/LV ≥ 1.0)**
CTR value that is the optimal cut-off point for prediction based on the ROC curve	>0.59	>0.55	>0.73	>0.73
sensitivity	0.882	0.488	1.000	1.000
specificity	0.385	0.750	0.077	0.083
accuracy	0.745	0.511	0.143	0.214
**D**	**Subgroup E: Men**		**Subgroup F: Women**	
	**Prediction of Right Ventricular Enlargement** **(RV/LV ≥ 0.9)**	**Prediction of Right Ventricular Enlargement** **(RV/LV ≥ 1.0)**	**Prediction of Right Ventricular Enlargement** **(RV/LV ≥ 0.9)**	**Prediction of Right Ventricular Enlargement** **(RV/LV ≥ 1.0)**
CTR value that is the optimal cut-off point for prediction based on the ROC curve	>0.59	>0.55	>0.58	>0.58
sensitivity	0.789	0.348	0.933	0.882
specificity	0.300	0.769	0.429	0.400
accuracy	0.538	0.487	0.773	0.773
**E**	**Subgroup M: Confirmed Pulmonary Embolism**		**Subgroup N: Excluded Pulmonary Embolism**	
	**Prediction of Right Ventricular Enlargement** **(RV/LV ≥ 0.9)**	**Prediction of Right Ventricular Enlargement** **(RV/LV ≥ 1.0)**	**Prediction of Right Ventricular Enlargement** **(RV/LV ≥ 0.9)**	**Prediction of Right Ventricular Enlargement** **(RV/LV ≥ 1.0)**
CTR value that is the optimal cut-off point for prediction based on the ROC curve	>0.62	>0.72	>0.54	>0.55
sensitivity	0.917	1.000	0.591	0.731
specificity	0.238	0.125	0.833	0.500
accuracy	0.485	0.576	0.643	0.714

CTR—cardiothoracic ratio; LV—left ventricle; ROC—receiver operating characteristic; RV—right ventricle.

## Data Availability

Study data can be made available upon documented request.
